# Theoretical–Experimental Study of the Action of Trace Amounts of Formaldehyde, Propionaldehyde, and Butyraldehyde as Inhibitors of the Ziegler–Natta Catalyst and the Synthesis of an Ethylene–Propylene Copolymer

**DOI:** 10.3390/polym15051098

**Published:** 2023-02-22

**Authors:** Joaquín Hernández-Fernández, Rodrigo Ortega-Toro, John R. Castro-Suarez

**Affiliations:** 1 Chemistry Program, Department of Natural and Exact Sciences, San Pablo Campus, University of Cartagena, Cartagena 130015, Colombia; 2 Chemical Engineering Program, School of Engineering, Universidad Tecnológica de Bolivar, Parque Industrial y Tecnológico Carlos Vélez Pombo Km 1 Vía Turbaco, Cartagena 130001, Colombia; 3 Department of Natural and Exact Science, Universidad de la Costa, Barranquilla 080002, Colombia; 4 Food Packaging and Shelf-Life Research Group (FP&SL), Food Engineering Department, Universidad de Cartagena, Cartagena de Indias 130015, Colombia; 5 Área Básicas Exactas, Universidad del Sinú, Seccional Cartagena, Cartagena 130001, Colombia

**Keywords:** formaldehyde–propionaldehyde and butyraldehyde, green ethylene, Ziegler–Natta, polypropylene, catalyst, degradation, random copolymer

## Abstract

The copolymer synthesis process can be affected by failures in the production process or by contaminating compounds such as ketones, thiols, and gases, among others. These impurities act as an inhibiting agent of the Ziegler–Natta (ZN) catalyst affecting its productivity and disturbing the polymerization reaction. In this work, the effect of formaldehyde, propionaldehyde, and butyraldehyde on the ZN catalyst and the way in which it affects the final properties of the ethylene-propylene copolymer is presented by analyzing 30 samples with different concentrations of the mentioned aldehydes along with three control samples. It was determined that the presence of formaldehyde 26 ppm, propionaldehyde 65.2 ppm, and butyraldehyde 181.2 ppm considerably affect the productivity levels of the ZN catalyst; this effect increases as the concentration of aldehydes is higher in the process; likewise, these impurities affect the properties of the final product, such as the fluidity index (MFI), thermogravimetric analysis (TGA), bending, tension, and impact, which leads to a polymer with low-quality standards and less resistance to breakage. The computational analysis showed that the complexes formed by formaldehyde, propionaldehyde, and butyraldehyde with the active center of the catalyst are more stable than those obtained by the ethylene-Ti and propylene-Ti complexes, presenting values of −40.5, −47.22, −47.5, −5.2 and −1.3 kcal mol^−1^ respectively.

## 1. Introduction

Ethylene (C_2_H_4_) is one of the essential olefins in petrochemical processes worldwide [1,2,3,4,5,6,7,8,9] produced industrially from an endothermic process based on cracking and pyrolysis of naphtha and light hydrocarbons [2,3]. Ethylene is considered one of the most manufactured products by the chemical industry, with approximately 75 million metric tons per year [1,2,3,4,5,6,7,8]. Its use is mainly related to its high energy density, the pi double bond, and the carbon content, which make it the ideal raw material for obtaining compounds of industrial interest, such as polymers, whether they belong to the families of homopolymers or copolymers [1,2]. Taking into account that ethylene is obtained from large amounts of petroleum and its production often leads to poor disposal of by-products such as carbon dioxide, toxic gases, and effluents [2,3,8], to the use of alternative sources for the production of ethylene has been considered, and this ethylene is called Green Ethylene (GE). It is a viable alternative used by the chemical industry to satisfy the great demand for ethylene worldwide and, at the same time, mitigate the environmental impact generated by its production from fossil fuels since both have the same properties and serve as raw material for the production of ethylene–propylene copolymers (CE-P) [6,7]. The GE is produced from the pyrolytic dehydration of ethanol, and this ethanol is derived from renewable sources such as corn, glucose, lignocellulosic biomass, etc. [2,4,5]. The production of ethylene–propylene copolymers is of great interest since it provides resins with marked properties, such as less brittleness and rigidity compared to pure polypropylene, which allows the production of materials with greater flexibility, elasticity, and a higher degree of resistance to impact [2,9,10,11]. The raw material with which CP-E is made is one of the most exciting points in the elaboration of these materials. In this process, we detect ZN catalysts, monomers (propylene, ethylene), cocatalyst (triethylaluminum, TEAL), selectivity agents and polymerization terminators (hydrogen) [2,4]. However, it is essential to strictly control the quality of the raw materials used during the synthesis of polymers since some components, such as the ZN catalyst, may be affected by the presence of traces of contaminating chemical compounds in the process. The presence of some chemical compounds such as ketones, hydrogen sulfide, arsine, and carboxylic acids can affect the production of CE-P since they act as inhibiting agents of the ZN catalytic systems by reacting with the active titanium (Ti) center of the catalyst, which prevents the formation of the Ti-Polypropylene complex generating delays or total loss of the polymerization process [12,13,14,15,16,17,18]. The final properties, such as MFI, thermal resistance, tensile strength, and elongation at the break of some olefins and polyolefins, are affected by the intervention of these impurities during the synthesis of the polymer. The inhibitor–Ti interaction is carried out through the interaction of the electropositive Ti with the free electron pair of the inhibitor, in which the electrons of the π bond of propylene predominate, therefore The formation of a π complex in Ti-propylene do not have barriers and are accompanied by a lower energy gain than that of the inhibitor–Ti, so the reaction with the inhibitor predominates. In the PP synthesis process, polymer chain propagation is carried out by moving propylene in Ti-PP, where the PP-alkyl chain has the olefin inserted. The growth of the PP chain is affected when the active site of Ti reacts with inhibitors of different polarities. The occurrence of these reactions depends on energetic factors [12,13,14,18,19]. Compounds such as Acetylene and Methylacetylene in concentrations from 0 to 40 ppm affect the productivity of the ZN catalyst since these poisons are adsorbed on the MgCl_2_ surface of the active center of the catalyst [19,20]. Catalyst productivity is expressed in metric tons per kilogram (MT/kg) and is understood as the MT of polymer produced for each kg of ZN catalyst used. Both experimental and computational studies allow us to determine the magnitude of the impact of these poisons and others not yet reported in the literature. To conduct investigations that will enable simulating these reactions in computational chemistry, calculations using density functional theory (DFT), electron configurations, and atomic geometry optimizations using Gaussian 09 software are required. The study of the impact of these impurities on the synthesis of CE-P is of great interest to the petrochemical industry since significant economic losses during the synthesis of these polymers due to low productivity, low physicochemical properties, excessive stoppages of industrial plants, etc., related to the presence of traces of these poisons. Previous investigations determined that compounds such as sulfur, unsaturated hydrocarbons, and permanent gases can affect the productivity of the ZN catalyst in the ranges of 5% to 22%, decreased thermal stability, increased melt flow rate, and affect molecular weight. This molecular weight has been determined experimentally and theoretically using the Bramner equation as a reference (Equation (1)) [13,16,17,18].

(1) $${\bar{M}}_{v}=\;-8480.6\;\times \;\mathrm{Ln}\;\mathrm{MFI}+62,836$$

In this research, 30 ethylene–propylene impact copolymers are synthesized using green ethylene as raw material. In this experiment, the impact of three concentrations of three chemical compounds named formaldehyde, propionaldehyde, and butyraldehyde on the productivity of the ZN catalyst is identified and quantified. Gas chromatography with mass spectrometry (GC-MS) is used to monitor the different concentration levels of these aldehydes during the synthesis. Thermogravimetric analysis (TGA) and melt flow index (MFI) are used to study the thermal stability behavior of the synthesized copolymers. To determine whether these aldehydes affect the mechanical properties of the copolymer, the tension, bending, and impact are quantified. After this experimental investigation and to understand the chemical reaction present in the experiment design, a reaction mechanism is proposed, and a computational evaluation is performed to corroborate the proposed mechanism.

## 2. Experimental

### 2.1. Materials and Methods

#### 2.1.1. Standards and Reagents

A commercial Ziegler–Natta (ZN) catalyst supported on MgCl_2_ (MgCl_2_/TiCl_4_, SINOPEC, Beijing, China) was used for synthesis. Airgas supplied ethylene (99.5% *w*/*w*) and propylene (99.7% *w*/*w*) monomers. Hydrogen was supplied by Linde, Colombia, with a concentration of more than 99.999%. The triethyl aluminum (TEAL) used as a cocatalyst was supplied by Albemarle Co. Dicyclopentyl dimethoxy silane (DCPDMS) as a Lewis base.

#### 2.1.2. Green Ethylene Production Process by Microwave-Assisted Ethanol Dehydration

The dehydration of ethanol to ethylene was carried out mainly in a microwave-assisted process. Two primary devices are used: a microwave-sensitive catalytic bed and its respective applicator. (1) A catalyst bed was prepared with ZSM-5/Zn/Mn zeolite granules and silicon carbide granules. This bed was with 8.2 g of ZSM-5 zeolite doped with 7.8 g of zinc and 0.15 g of manganese. (2) A total of 20 g/min of ethanol was fed to a vaporizer until it reached its boiling point. (3) A flow of N_2_ was injected to entrain the steam, and (4) the N_2_-ethanol steam mixture was taken to a reaction chamber with a microwave-sensitive catalytic bed.

#### 2.1.3. Copolymer Synthesis Process Using Green Ethylene as a Sustainable Feedstock

The synthesis was carried out in a stainless-steel reactor with a 2 L capacity and the polymerization was carried out at 60 °C to guarantee the pre-polymerization of the PP. The TEAL cocatalyst was added to the reactor with an Al/Ti ratio of 100. This ratio helps to release the active sites of the ZN catalyst and improves the morphology of the polymer gram. After 20 min, hydrogen was injected to ensure that the polymer chains have equal chain lengths. Then, ethylene green was injected at 0.6 MPa to initiate the homopolymerization of the ethylene green in suspension for 30 min. During the synthesis, approximately 50 mg of ZN-catalyst was added, the solution was saturated with propylene at 0.1 MPa, mechanical stirring was carried out at 300 rpm and propylene was continuously added at 1 atm of pressure. This copolymer was synthesized using the proportions of ethylene and propylene of 25 and 75%, respectively.

**Figure 1:**
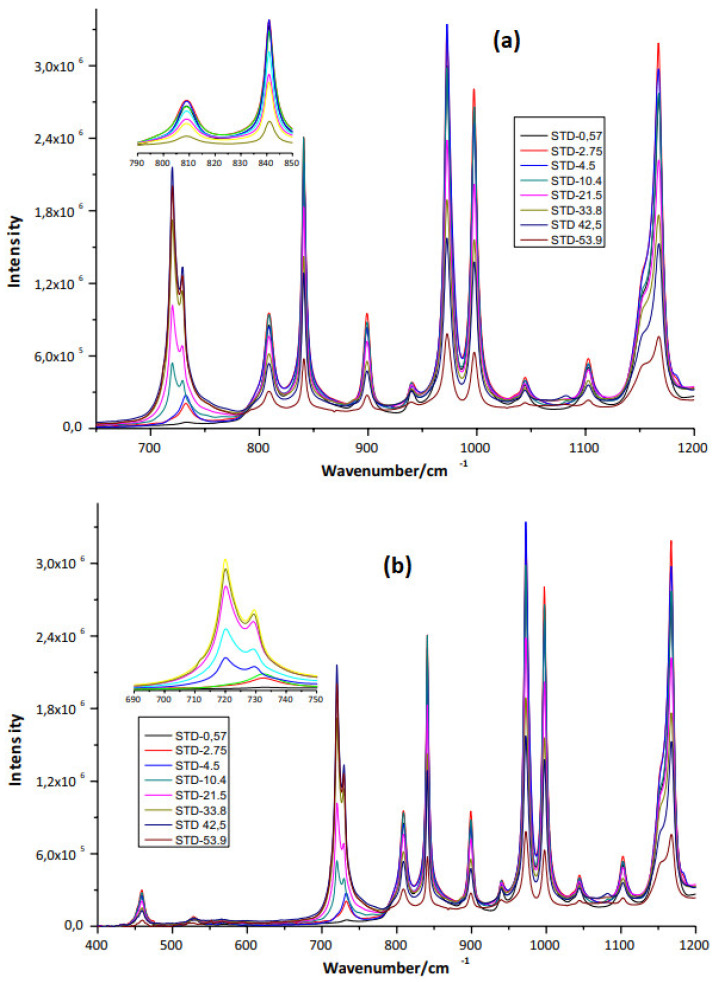
Infrared spectra of ethylene standards in ethylene-propylene impact copolymers. (**a**) Between 600–1200. (**b**) 400–1200.

Eight ethylene-propylene copolymer standards with different ethylene concentrations were used. The ethylene concentrations were 0.57%, 2.75%, 4.5%, 10.4%, 21.5%, 33.8%, 33.8%, 42.5%, and 53.9%. The infrared spectra of each standard are shown in Figure 1a,b. With this information, a calibration curve has been created that has allowed us to determine the experimental concentration of ethylene within the copolymer samples synthesized in this investigation. This copolymer was synthesized using the proportions of ethylene and propylene of 25 and 75%, respectively.

The structure of the copolymer macromolecule has a linear chain of C–C and C–H moiety (with some branches) in different conformations, which are based on C–H sequences. This forms the structure of the ethylene-propylene copolymer. In Figure 1a,b, the IR spectra, as expected, are pretty simple and consist of bands mainly corresponding to different modes of vibration of C–C and C–H bonds. The IR spectrum in the range of 1000–4000 cm^−1^ (Figure 1a,b) for the copolymer standards shows the characteristic absorption bands mainly in 2900 cm−^1^ (C–H stretch) and 1460–1165 cm^−1^ (C–H flexion and stretching C–C). These modes of vibration are highly variable in terms of energy absorption and the chemical environment of each functional group or atomic/molecular arrangements within the polymer matrix. The samples showed similar spectra containing the same number of bands without shift, but the intensity of the bands was different for each type of PP (Figure 1a,b). The spectra observed include bands at 810 cm^−1^ (C–C stretching), 841 cm^−1^ (C–CH_3_ rocking), 900 cm^−1^ (CH_3_ stretching), 974 cm^−1^ (CH_3_ rocking), 999 cm^−1^ (CH_3_ rocking), 1152 cm^−1^ (C–C stretching), 1220 cm^−1^ (CH_2_ twisting), 1329 cm^−1^ (CH_2_ twisting), 1359 cm^−1^ (CH_2_ wagging), 1436 cm^−1^ (CH_2_ bending), and 1459 cm^−1^ (CH_2_ bending). The spectra of the homo PP pellet showed a higher intensity band between 200 and 1220 cm^−1^ than impact PE-PP and random PE-PP copolymers (Figure 1a,b). The molecular structure of impact PE-PP and random PE-PP could explain this behavior. Thus, the homo PP contains major quantity of CH_3_ groups and could explain the higher intensity of this band in the spectral region below to 1220 cm^−1^. Principal vibrational bands observed correspond to assigned CH_2_ rocking and C–CH_3_ stretching at 841 cm^−1^, assigned CH_3_ rocking at 974 and 999 cm^−1^, and assigned CH_3_ stretching at 900 cm^−1^. In addition, this region is characterized by rich skeletal vibration information.

#### 2.1.4. Gas Chromatography with Selective Mass Detector (GC-MSD)

The quantification process was carried out with the help of a gas chromatograph (Agilent 7890B), which has a front injector and a rear injector (250 °C, 7.88 psi, 33 mL min^−1^) (250 °C, 11.73 psi, 13 mL min^−1^) respectively. The oven was started at 40 °C × 3 min, increased to 60 °C at 10 °C min^−1^ × 4 min, and then increased to 170 °C at 35 °C min^−1^ [19]. The working volume varied between 0.25 and 1.0 mL; this depended on the circuit of each valve.

#### 2.1.5. The Melt Flow Index (MFI)

A Tinius Olsen MP1200 plastometer was used to determine the MFI. The working temperature inside the equipment cylinder was 230 °C, and a 2.16 kg piston was used to move the molten material.

#### 2.1.6. Thermogravimetric Analysis (TGA)

The determination of the thermal degradation of the samples was performed with the help of a thermal analyzer TGA Q500 (TA Instruments). The heating process was carried out at 10 °C min^−1^ from 40 to 800 °C in an air atmosphere (50 cm^3^ min^−1^) [19].

#### 2.1.7. Fourier Transform Infrared (FTIR)

FTIR was used to measure the structural changes in the polymeric matrix, since, when exposed to high temperatures (400 °C), a thermal degradation of the matrix occurs. A Nicolet 6700 FTIR (Thermo Scientific, Waltham, MA, USA) was used.

#### 2.1.8. Mechanical Properties of Copolymer

To determine the mechanical properties of the copolymer, composites based on thermoplastic sources should be worked with the help of a twin-screw extruder and then subjected to an injection molding process. Test specimens were prepared according to ASTM standards, and two molds were used to prepare tensile (ASTMD638) and flexural (ASTMD790) test specimens. The mold temperature was maintained at 50 °C. During the tensile test, a uniaxial tensile load is applied to both ends of the specimen. After placing the specimen in clamps, the electromechanical system stretches the material vertically. ASTMMD638 performed the tensile test on a computerized universal testing machine H50KL (TiniusOlsen). Considering that the specimens were produced by injection molding, the specimens were classified as TYPE I specimens. The calibrated length value (G) of the test specimens was 50 mm, the narrow section width (W) was 12.7 mm, and the thickness (T) was 3.4 mm.

The flexural strength was evaluated with the aid of H50KL (TiniusOlsen) equipment, according to ASTMD790. The Izod impact test was performed with an impact pendulum model IT504 (TiniusOlsen) according to ASTMD256. For this purpose, one end of the specimen was fixed with a cantilevered vise. Each specimen was assigned a 45° and 2.5 mm deep AV notch.

Ead = EMg/P − EMg − EP.
(2)

#### 2.1.9. Computational Details to Study the Reaction of the ZN Catalyst with Formaldehyde, Propanaldehyde and Butyraldehyde Residues

The vibrational analysis was performed under controlled conditions (P = 1 Atm and T = 298 K) in order to calculate the enthalpy (Had) and Gibbs free energy [15]. All calculations were performed with the help of density functional theory (DFT); the Gaussian 09 software helped to determine the geometrical optimality along with the electronic configuration [15], the Perdew functional and Ernzerhof (PBEh1PBE) [21] using an ensemble augmented by a polarization function (Gaussian basis set TZVP) [22]. Unconstrained calculations are performed with triplet and quintet spin states for the case of Ti (III) species and those containing O_2_ molecules, respectively.

## 3. Results and Discussion

### 3.1. Effects of Traces of Formaldehyde, Propianoldehyde and Butyraldehyde on the Polymerization Process of Random Copolymer Rat

**Figure 2:**
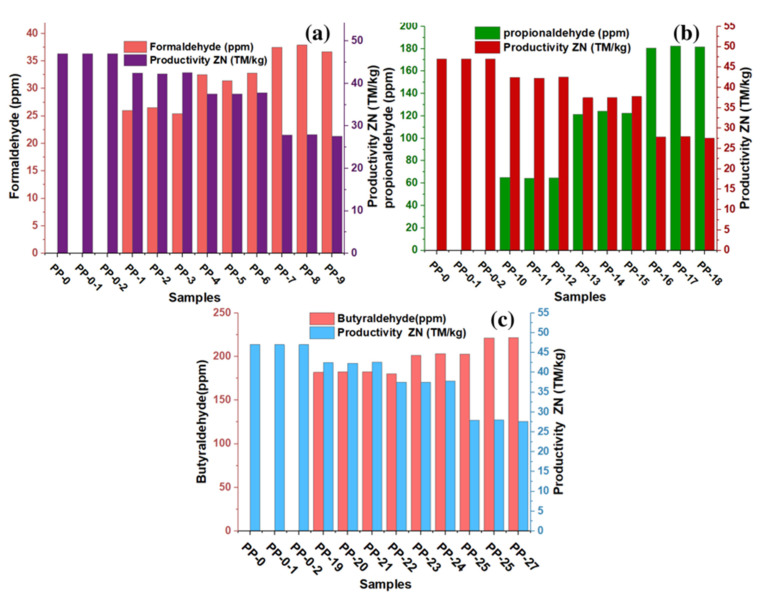
Effect of trace formaldehyde (**a**), propionaldehyde (**b**), and butyraldehyde (**c**) on the efficiency of copolymer synthesis.

The productivity losses of the ZN catalyst during the production of polypropylene or copolymers are linked to the interaction that impurities have with the active center Ti, thus inhibiting polymerization during the process, which prevents obtaining the desired amount of product. It should be noted that the inhibitory capacity of these impurities on the ZN catalyst has been previously reported [16,19]. In this experimentation, the impact of these aldehydic derivatives on the productivity of the ZN catalyst has been evaluated. These aldehydes are characterized by having a carboxyl group in their molecular structure, which must react with the active center of the catalyst. In Figure 2a–c of this experiment, it can be seen that the increase in the concentration of formaldehyde, propionaldehyde, and butyraldehyde produces a decrease in catalyst productivity. In the absence of these aldehydes, the productivity of the ZN catalyst was 47 MT/kg. In Figure 2a–c, productivity losses of 10% are observed for formaldehyde concentrations between 25.4 and 26.5 ppm, for propionaldehyde concentrations they are between 64.2 and 65.5 ppm, and for butyraldehyde concentrations they are between 181.8 and 182.4 ppm. They maintain that the aldehyde with higher polarity, lower number of carbon atoms, and lower molecular weight has required lower concentration to decrease productivity from 47 MT/kg to approximately 42 MT/kg. Productivity losses of 20% were observed when formaldehyde concentrations between 31.4 and 32.8 ppm, propionaldehyde concentrations between 64.2 and 62.2 ppm, and butyraldehyde concentrations between 200 and 203.4 ppm were added independently in the reactor. In the research, the most significant productivity losses were 41%; in this scenario, the ZN catalyst productivity dropped to approximately 27.6 MT/kg, and the highest levels of formaldehyde, propionaldehyde and butyraldehyde that caused these losses were 36.7, 181.6, and 221.5 ppm, respectively.

### 3.2. Computational Study of Formaldehyde, Propionaldehyde and Butyraldehyde Interaction with the Active Center of Ti (Poison-Ti Interaction)

This quantum mechanical calculation model was chosen since it shows that the relationship of TiCl_4_ with the geometric plane (104) is relatively weak and is favored in energetic terms [23]. These energetic events are of great importance for this research since we intend to understand the ways in which the reactions of these aldehydes with the ZN catalyst can occur, propose a reaction mechanism, and understand the ways in which this can affect the properties of these copolymers. The reactions of formaldehyde, propionaldehyde and butyraldehyde with the center of the titanium (Ti) atom of the ZN catalyst on the MgCl_2_ surface are favored by −40.5, −47.22, and −49.75 kcal mol^−1^, respectively. In this paper, it was indicated that the reactivity of these aldehydes increases with polarity and decreases with the increase in the length of the carbon numbers in these aldehydes. So, the formaldehyde reactivity through the center of the Ti atom is greater than that of propionaldehyde and butyraldehyde. The binding between these aldehyde impurities and the active Ti of the ZN catalyst is very favorable compared to the other interactions studied (see Ead data in Table 1). Table 1 shows that the chemical complexes formed by ethylene-Ti and propylene-Ti are stable only at 1.3 and 5.2 kcal mol^−1^, respectively [15]. This indicates that the reactions of Ti with the Aldehyde impurities are more visible, given their excellent stability. Therefore, this explains the decrease in productivity of the catalyst with the presence of aldehydes and also the potential of the loss of productivity to reach 40% with only the presence of small amounts of these aldehydes.

**Figure 3:**
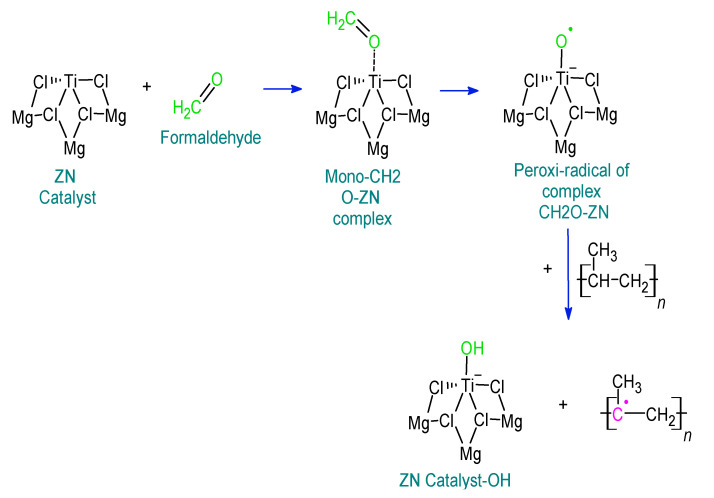
Effect of formaldehyde, propionaldehyde, and butyraldehyde traces on the efficiency of the synthesis of the copolymer.

The presence of formaldehyde, propionaldehyde, and butyraldehyde directly affects the synthesis of polypropylene and, therefore, the production of the copolymer. This affectation occurs mainly in the polymerization phase of the production of these olefins where the capacity of the catalyst agent is inhibited. Based on Table 1, a reaction mechanism of these impurities with the ZN catalyst is proposed, as shown in Figure 3.

During the synthesis of copolymers, chain propagation is one of the most critical steps; propylene interacts with Ti to form Ti-PP [24,25,26,27,28,29,30,31,32]. This process is affected by the participation of chemical molecules, which act as inhibitors of the ZN catalyst, which depends on certain energetic factors, as indicated in Table 1. Then, the reactive face of Ti is occupied by these inhibitors, avoiding the ethylene binding, and propylene results in the non-growth of the polymeric chain [13]. If the poisoning of the ZN catalyst is complete, there will be no possibility of catalyst–propylene and catalyst–ethylene interaction.

### 3.3. Effects of Formaldehyde, Propionaldehyde and Butyraldehyde on the Thermal Properties of the Random Copolymer

**Figure 4:**
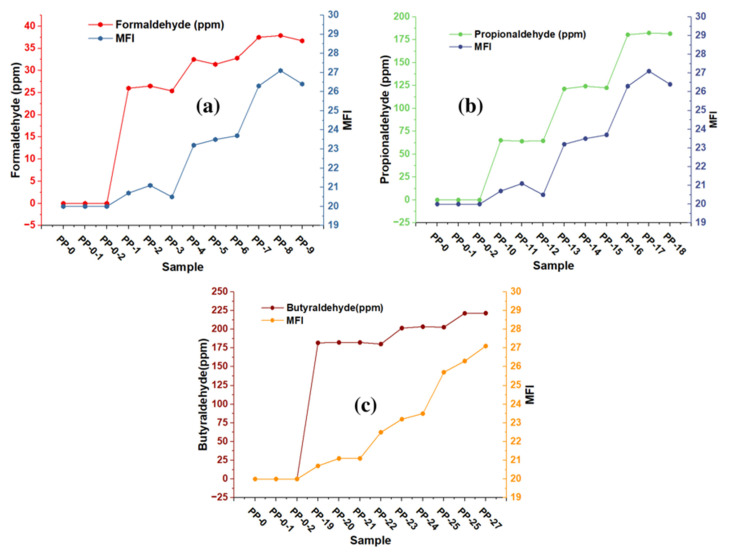
Effect of trace formaldehyde (**a**), propionaldehyde (**b**), and butyraldehyde (**c**) on the MFI of the copolymer.

The thermal properties of the copolymer are one of the most critical qualities for both polymer processing and for evaluating multiple changes that can affect the quality of polymers during application [25,26,27,28,29,30]. With MFI, we can estimate melt processing since the higher the MFI values, the higher the melt flowability, and the lower the MFI values, the lower the polymer melt flowability. MFI measurements on each copolymer sample were performed in triplicate for each aldehyde concentration present, showing RSD values between 1.07 and 2.63. The MFI results are shown in Figure 4a–c and it can be observed that the MFI increases with the presence of aldehydes in the green ethylene used as raw material in the production of the copolymer. Formaldehyde concentrations (between 25.4 and 26.5 ppm), propionaldehyde concentrations (between 64.2 and 65.5 ppm), and butyraldehyde concentrations (between 181.8 and 182.4 ppm) produce MFIs of approximately 21, which shows a significant increase in the fluidity of these samples. Figure 4a–c show that formaldehyde (between 31.4 and 32.8 ppm), propionaldehyde (between 64.2 and 62.2 ppm) and butyraldehyde (between 200 and 203.4 ppm) produce MFI with approximate values of 23. Since we are able to visualize the increase in the IMF in 3 units, we can observe that the highest MFI values ranged from 26 to 27, obtained with the highest concentrations of aldehydes (formaldehyde with 36.7 ppm, propionaldehyde with 181.6 ppm, and butanaldehyde with 221.5 ppm).

It is essential to highlight that the inhibitory effect of the presence of impurities in synthesizing resins with industrial interest has already been reported previously (Hernandez et al., 2022). They demonstrated that compounds such as arsine in concentrations of 0.05 to 4.73 affect the MFI and the molecular weight of the samples studied.

**Figure 5:**
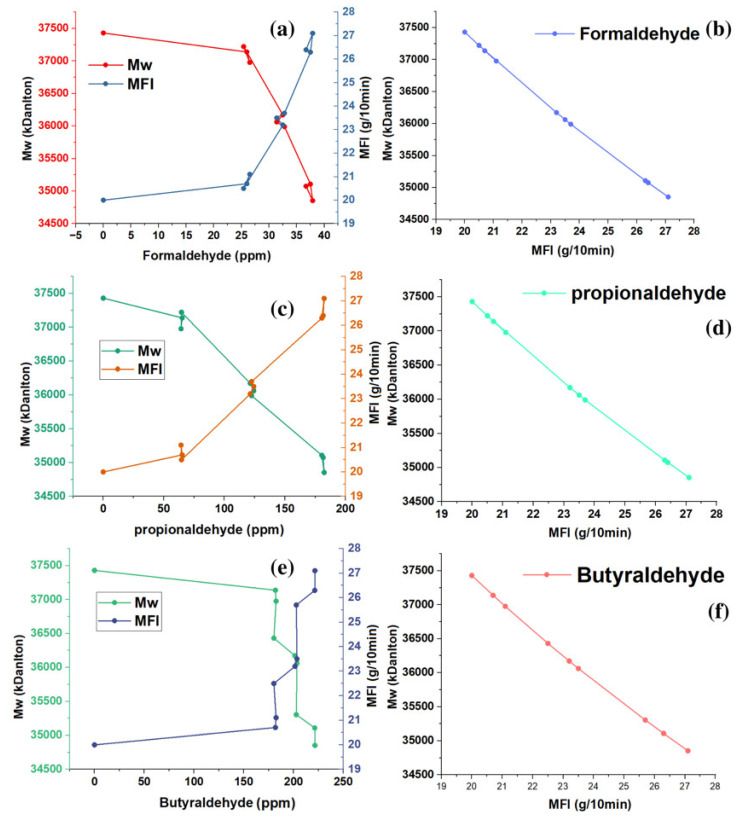
Effect of trace formaldehyde (**a**,**b**), propionaldehyde (**c**,**d**), and butyraldehyde (**e**,**f**), on the MFI and Mv of the copolymer.

The Bramner equation shows the relationship between the MFI and average molecular weight (Mw). In Figure 5a–f, an inverse relationship is observed between the MFI and the Mw. The samples with MFI of 20 g/10 min appeared with Mw that oscillated around 37,430 kDalton. MFIs between 20.5 and 21.1 g/10 min showed Mw between 37,220 and 36,976 kDaltons. The samples of the copolymers with the highest MFI values ranged between 25.7 and 27.1 g/10 min, with Mw that ranged between 35,304 and 34,854 kDaltons.

### 3.4. Effect of Trace Formaldehyde, Propionaldehyde and Butyraldehyde on the TGA of the Copolymer

**Figure 6:**
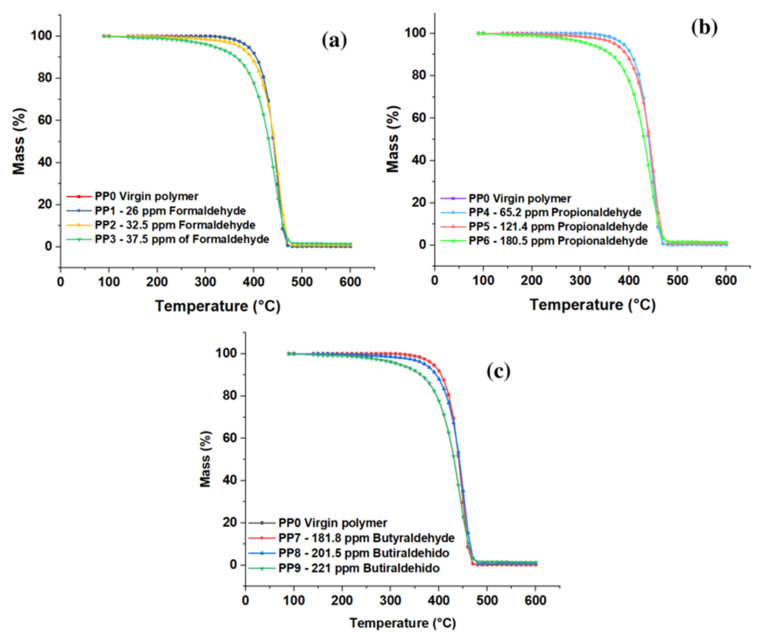
Effect of trace formaldehyde (**a**), propionaldehyde (**b**), and butyraldehyde (**c**) on the TGA of the copolymer.

The thermal resistance of the copolymer obtained with varying concentrations of formaldehyde, propionaldehyde, and butyraldehyde was acquired by thermogravimetric analysis (TGA, Figure 6a–c); the analysis was performed in a temperature range from 90 °C to 600 °C, thus identifying the percentage of weight loss in each sample. In the case of samples PP1, PP4, and PP7, whose aldehyde concentration was 26 ppm formaldehyde, 65.2 propionaldehyde, and 181.8 butyraldehyde, respectively, there were no significant differences in thermal degradation concerning PP0 (blank sample). On the other hand, a second group formed by samples PP2, PP5, and PP8 showed similar behavior in the degradation process, where the data obtained show that they presented weight loss starting at 100 °C and reached a weight loss of 50% once the temperature of reached 440 °C; finally, these samples showed a total weight loss at 470 °C. Samples PP3, PP6, and PP9 showed a weight loss of 50% at 430 °C and 98% at 540 °C.

These results show that the concentration of impurities such as aldehydes directly affects the thermal stability of the copolymer since it is observed that as the amount of formaldehyde (See Figure 6a), propionaldehyde (See Figure 6b), and butyraldehyde (See Figure 6c) in the resin increases, its thermal properties decrease; this is due to the inhibitory effect of aldehydes in the polymerization process [12]. The presence of impurities in the polymerization process prevents complete polymerization, which generates new compounds that alter the macromolecular composition of the material [30,31,32,33,34,35,36,37,38].

Hernandez et al., 2022, showed that compounds such as traces of sulfhydric acid in concentrations of 0.0 to 5 ppm affect properties such as TGA in synthesizing resins in lower temperature ranges concerning aldehyde molecules. In the case of sulfhydric acid, losses of 50% were observed at 205 °C at its highest concentration, while for aldehydes, these values were obtained at 440 °C.

### 3.5. Effects of Formaldehyde, Propionaldehyde and Butyraldehyde on the Random Copolymer’s Mechanical Properties (Tensile, Flexural, and Impact)

**Figure 7:**
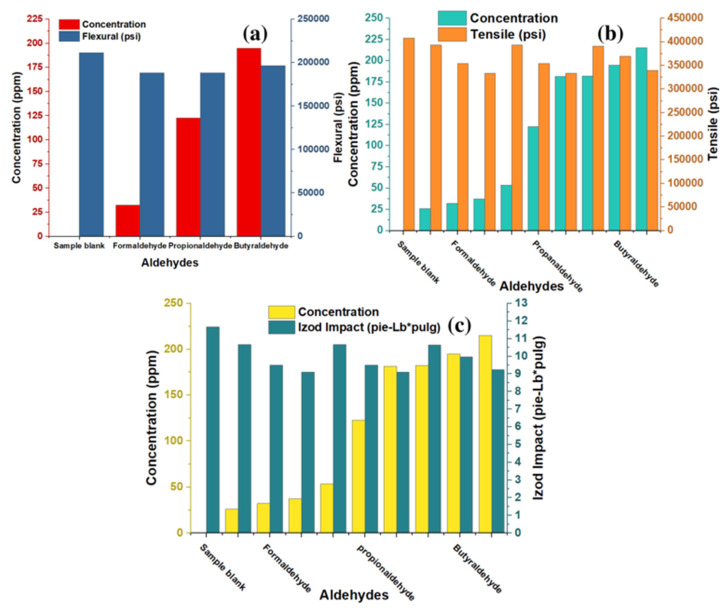
Effects of formaldehyde, propionaldehyde, and butyraldehyde on the random copolymer’s mechanical properties. Flexural (**a**), tensile (**b**) and Izod Impact (**c**).

To determine the incidence of aldehydes on the mechanical properties of the copolymer, tension, bending, and impact of each of the samples under study were evaluated. The results are illustrated in Figure 7a–c for flexural, tensil and izod impact; they show that the concentration of aldehydes has an indirectly proportional relationship with the mechanical properties since as the aldehyde shrinkage increases, the tension, bending, and impact values decrease. For the impact test, it is observed that the reference or blank sample obtained values of 12 ft-Lb*in; the values closest to this were obtained in those samples with a lower concentration of aldehydes; for the case of formaldehyde, values of 11 ft-Lb*in in those samples with 26 ppm of the compound were acquired. In contrast, this same value was achieved with samples of 53 and 182 ppm propionaldehyde and butyraldehyde, respectively. However, the samples with 37 ppm concentration of formaldehyde obtained a value of 9 ft-Lb*in, with 182 ppm of propionaldehyde and 215 ppm of butyraldehyde presenting the exact value of 9 ft-Lb*in, this being the lowest data shown in all samples (see Figure 5).

In the data obtained during the flexural and tensile test for the samples with formaldehyde, propionaldehyde, and butyraldehyde, the behavior does not vary concerning those obtained in the impact test, where a higher result is obtained in the samples with a lower concentration of aldehydes and followed by a decrease in the data of those with a higher presence of impurities in their composition. In the case of formaldehyde, data of 213,076.33 psi in bending and 393,333 psi in tension were obtained in the samples with 26 ppm of the compound. In comparison, the samples with 37 ppm obtained lower results showing 172,785 psi in bending and 333,832.33 psi in tension; this same behavior is observed for propionaldehyde and butyraldehyde.

The results obtained at the mechanistic level are directly related to those presented in terms of TGA and MFI, where it is evident that the presence of aldehydes can affect the behaviour of the properties as the concentration of aldehydes increases in the CE-P, indicating the presence of a smaller number of propylene chains. Hernandez et al., 2022, showed that arsenic at concentrations of 0.1 to 3.0 ppm affects the integration of the complex formed during polymerization, which leads to more ends being inserted into the structural chains and causes a decrease in molecular weight, increasing the MFI and reducing the ability to resist stress, which generates failures and that the resins obtained break with lower elongation [19,24,25].

## 4. Conclusions

Formaldehyde concentrations (between 25 and 38 ppm), propionaldehyde (between 64 and 182 ppm), and butyraldehyde (between 182 and 222 ppm) decreased catalyst productivity between 10 and 40%, increased MFI between 3.8 and 25%, decreased flexural between 0.7 and 18%, decreased the tensile between 2 and 19%, decreased the impact between 8 and 21%. The computational chemistry studies allow us to determine that the aldehyde–titanium interactions are more stable than the propylene–titanium and ethylene–titanium interactions. They have shown that the theoretical and experimental results are complementary to understanding the impact of these inhibitors during the synthesis of these copolymers.

## Figures and Tables

**Table 1:** Binding energies, Ead, in kcal mol^−1^, for the interaction of different poisons with Ti and Mg in cluster.

Computational Data	ZN Inhibitors			Monomers	
	Formaldehyde	Propionaldehyde	Butyraldehyde	Propylene	Ethylene
(kcal mol^−1^)	−40.5	−47.22	−49.75	−5.2	−1.3
Ead ^b^	−31.5	−39.6	−41.7	--	--
Had ^b^	−25.6	−33.4	−35.1	--	--
Gad ^b^	−24.5	−30.2	−35.2	--	--

For the interaction of different poisons with Ti in Mg_9_Cl_18_-Ti_3_ Cl_2_CH_3_ cluster. ^b^ for the interaction of different poisons with Mg in TiIIICl_2_CH_3_-Mg_16_Cl_32_ cluster.

## Data Availability

Not applicable.
